# Social Hierarchy Dictates Intestinal Radiation Injury in a Gut Microbiota-Dependent Manner

**DOI:** 10.3390/ijms232113189

**Published:** 2022-10-29

**Authors:** Xiaozhou Zeng, Zhihong Liu, Yanxi Dong, Jiamin Zhao, Bin Wang, Huiwen Xiao, Yuan Li, Zhiyuan Chen, Xiaojing Liu, Jia Liu, Jiali Dong, Saijun Fan, Ming Cui

**Affiliations:** 1 Tianjin Key Laboratory of Radiation Medicine and Molecular Nuclear Medicine, Institute of Radiation Medicine, Chinese Academy of Medical Sciences & Peking Union Medical College, Tianjin 300192, China; 2 Department of General Surgery, The Second Affiliated Hospital of Soochow University, Suzhou 215123, China; 3 Department of Microbiology, College of Life Sciences, Nankai University, Tianjin 300071, China

**Keywords:** social hierarchy, radiotherapy, radiation-induced intestinal toxicity, gut microbiota, probiotics

## Abstract

Social hierarchy governs the physiological and biochemical behaviors of animals. Intestinal radiation injuries are common complications connected with radiotherapy. However, it remains unclear whether social hierarchy impacts the development of radiation-induced intestinal toxicity. Dominant mice exhibited more serious intestinal toxicity following total abdominal irradiation compared with their subordinate counterparts, as judged by higher inflammatory status and lower epithelial integrity. Radiation-elicited changes in gut microbiota varied between dominant and subordinate mice, being more overt in mice of higher status. Deletion of gut microbes by using an antibiotic cocktail or restructuring of the gut microecology of dominant mice by using fecal microbiome from their subordinate companions erased the difference in radiogenic intestinal injuries. *Lactobacillus murinus* and *Akkermansia muciniphila* were both found to be potential probiotics for use against radiation toxicity in mouse models without social hierarchy. However, only *Akkermansia muciniphila* showed stable colonization in the digestive tracts of dominant mice, and significantly mitigated their intestinal radiation injuries. Our findings demonstrate that social hierarchy impacts the development of radiation-induced intestinal injuries, in a manner dependent on gut microbiota. The results also suggest that the gut microhabitats of hosts determine the colonization and efficacy of foreign probiotics. Thus, screening suitable microbial preparations based on the gut microecology of patients might be necessary in clinical application.

## 1. Introduction

Social dominance hierarchy is a common phenomenon among social animals [1,2,3,4]. Social hierarchies determine the quantity and quality of resources, profoundly influencing the survival, health, reproduction and other behaviors of social animals [5,6]. Social hierarchy is associated with a variety of physiological and biochemical responses. For instance, group members of different social status accordingly exhibit different hormone levels, urinary protein content, and body temperature regulation [5,7,8,9]. Aggressive behavior among animals is a connatural heritable trait contributing to the establishment of social hierarchy [3,10,11,12]. Although animals with higher social status gain more resources through competition, they also suffer more unavoidable stress, physical danger, and metabolic demands [13]. The understanding of the relationship between social hierarchy and stress reaction remains limited, especially regarding non-social stress such as oxygen deficit, drug intervention, and radiation exposure [14].

Iatrogenic and accidental radiation exposure cause progressive and intractable injuries. Although advancement in delivery technology has transformed radiation therapy into a more precise treatment, its use as a cancer treatment inevitably harms the neighboring tissues and organs owing to proximity, radiation leakage, etc., resulting in multiple adverse events on patients [15]. Intestinal radiation syndrome is one of the major side effects connected with abdominal and pelvic radiotherapy [16]. The stress response of an organism to these complications depends on complex substances in gastrointestinal tract [17,18,19]. The human body harbors trillions of microorganisms, with their population reaching the highest density in the intestinal compartment where they form a sophisticated microecology termed as the gut microbiome [20]. The gut microbiome is a key regulator for the immune systems and metabolic processes of hosts to maintain body health [21,22,23,24]. Recent studies have reported that gut microbiota are implicated in the social hierarchy formation of animals [25,26,27]. Mice in the same colony with different social dominance status carry characteristic configurations of gut microbiota, while the gut microbiota metabolites are able to influence the establishment of social hierarchy [25]. Our previous studies identified that the gut microbiome is involved in radiation-induced toxicity and could be employed to combat acute radiation syndrome in mouse models without social dominance hierarchy [28,29,30,31,32,33]. However, it remains poorly understood whether social hierarchy impacts the development of radiation toxicity.

In this study, we aimed to investigate the effects of social hierarchy on intestinal radiation injury and uncover the underlying mechanism. We observed that mice in higher ranks of social hierarchy exhibited more serious intestinal radiation toxicity compared with their counterparts of lower status. Our findings provide novel insights into the development of radiation injury in natural habitats and pave a way to protect against intestinal radiation toxicity in a pre-clinical setting.

## 2. Results

### 2.1. Social Hierarchy Determines the Degree of Intestinal Radiation Injury

**Figure 1:**
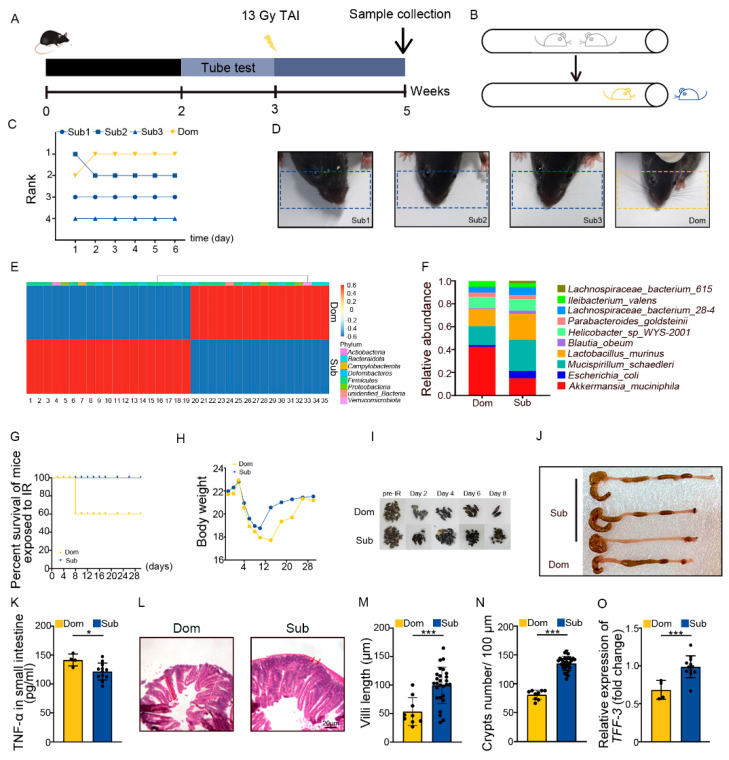
Social hierarchy determines the degree of intestinal radiation injury. (**A**) Experimental protocol. (**B**) Schematic of the tube test. (**C**) The ranking positions of one cage of mice tested daily over 6 days. (**D**) Photographs of barber and receptor mice. (**E**) Differences in gut microbiota at the genus level between Dom and Sub mice were assessed by 16S rRNA. Details of the bacteria are listed in Supplementary Table S2. (**F**) The relative abundance of the top 10 different strains of bacteria at the genus level in mice of the two groups. (**G**) Survival rate of Dom and Sub mice after irradiation. (**H**) Body weight of mice after exposure to irradiation. (**I**) Photographs of formed fecal pellet from experimental mice before and after irradiation. (**J**) Photographs of dissected colons from mice in the two groups. (**K**) Protein levels of TNF−α in small intestines were measured by ELISA. (**L**) Morphology of the small intestine was shown by H&E staining. (**M**) Length of small intestinal villi in Dom and Sub mice. (**N**) The number of small intestinal crypts in Dom and Sub mice. (**O**) The mRNA expression levels of *TFF−3* in small intestine tissues from Dom and Sub mice were examined by qRT−PCR. Statistically significant differences are indicated: Student’s *t* test, n = 4 in Dom group, n = 12 in Sub group, * *p* < 0.05 and *** *p* < 0.001.

The tube test is a robust and reliable behavioral assay to investigate social hierarchy in mice [34]. Four mice were co-housed for 2 weeks then were tested pair-wise (Figure 1A,B). Based on the tube test, mice were categorized as one dominant (Dom) mouse and three subordinate (Sub) mice in a cohort (Figure 1C, Figure S1A,B). The results of the tube test were validated by barber assay (Figure 1D). We performed 16S rRNA sequencing to assess the gut microbiota, and observed that mice of different social hierarchical status harbored specific intestinal bacteria configurations (Figure 1E,F, Supplementary Table S2), which was in line with the previous study [26]. Then, the Dom and Sub mice were exposed to total abdominal irradiation (TAI) to mimic radiotherapy for pelvic and abdominal cancers. Intriguingly, Dom mice showed a lower survival rate following 18 Gy of TAI (Figure 1G). Dom mice also experienced overt intestinal radiation toxicity, as judged by lower body weight (Figure 1H) and food intake (Figure S1C), fewer formed fecal pellets (Figure 1I, Figure S1D), shorter colons (Figure 1J, Figure S1E), and higher levels of inflammatory factors (Figure 1K, Figure S1F–J). H&E staining showed that the intestinal villi were scarcer and shorter, and the crypts were less frequent in Dom mice than in Sub ones (Figure 1L–N). In parallel, qRT-PCR assay further revealed lower expression levels of intestinal integrity-related genes in the small intestines of Dom mice (Figure 1O, Figure S1K,L). All the evidence indicates that mice of higher status were more predisposed to serious intestinal radiation injuries compared with the ones of low ranking.

### 2.2. Irradiation Molds Characteristic Gut Microbiota Signature between Dom and Sub Mice

**Figure 2:**
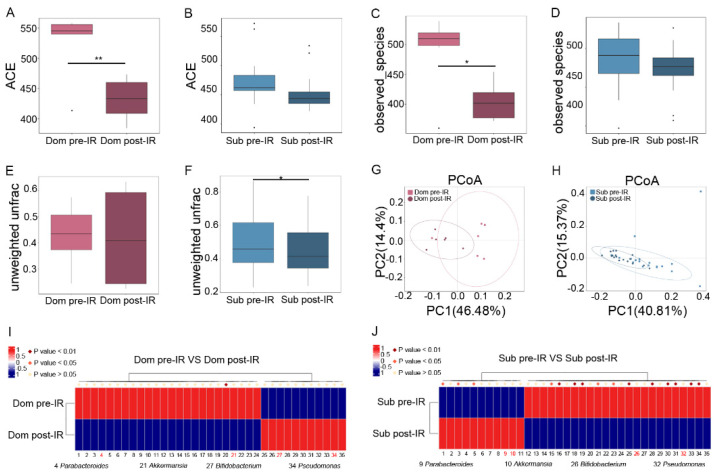
Irradiation affects characteristic gut microbiota signatures in Dom and Sub mice. The α−diversity of enteric bacteria of Dom and Sub mice before and after radiation was compared by (**A**,**B**) ACE index and (**C**,**D**) observed species. The β−diversity of enteric bacteria of (**E**) Dom mice and (**F**) Sub mice was compared by unweighted unifrac analysis. (**G**,**H**) PCoA was applied to examine the alteration of intestinal bacteria taxonomic patterns. (**I**) Differences of gut microbiota in Dom mice before and after 13 Gy TAI. (**J**) Differences of gut microbiota in Sub mice before and after 13 Gy TAI. Details of the bacteria are listed in Supplementary Table S3. Statistically significant differences are indicated: Student’s *t* test, n = 6 in Dom pre−IR and Dom post−IR groups, n = 18 in Sub pre−IR and Sub post−IR groups, * *p* < 0.05 and ** *p* < 0.01.

To reveal the mechanism of different responses of hierarchic mice to irradiation, we analyzed the gut microbiota profile in Dom and Sub mice before and after 13 Gy TAI. Radiation exposure introduced a notable reduction in α-diversity of fecal pellets from Dom mice, while the changes in Sub mice were negligible (Figure 2A–D, Figure S2A–D). Although unweighted unifrac analysis showed a slight decrease in β-diversity of gut microbiota from Sub mice (Figure 2E,F), principal coordinates analysis (PCoA) revealed a visible separation of gut bacteria signatures in Dom mice following irradiation (Figure 2G,H). These results suggest that the gut microbiota in Dom mice were susceptible to radiation stimuli. In detail, local abdominal irradiation reduced the relative abundance of Parabacteroides and Akkermansia at the genus level in the Dom group, and elevated their levels in Sub mice (Figure 2I,J, Supplementary Table S3). In contrast, the relative abundance of Pseudomonas and Bifidobacterium at the genus level was decreased in Sub mice after radiation, and was increased in the Dom group (Figure 2I,J, Supplementary Table S3). Together, our observations showed that gut microbiota exhibited different alteration paradigms between Dom and Sub mice following radiation exposure.

### 2.3. Social Hierarchy Impacts the Development of Intestinal Radiation Injuries in a Gut Microbiota-Dependent Manner

**Figure 3:**
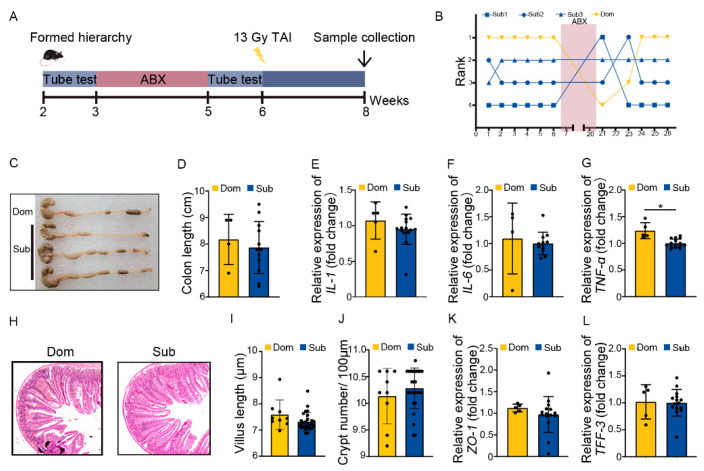
ABX treatment eliminated the difference in intestinal radiation injury between Dom and Sub mice. (**A**) Experimental protocol. (**B**) The ranked positions of one cage of mice tested daily before and after ABX treatment. (**C**) Photographs of dissected colons from mice in the two groups. (**D**) Statistical results of colon length between the two groups. (**E**–**G**) The mRNA levels of *IL−1*, *IL−6*, and *TNF−α* in small intestinal tissues of mice were examined by qRT−PCR. (**H**) The morphology of small intestine was revealed by H&E staining. (**I**) Length of small intestinal villi in Dom and Sub mice. (**J**) Numbers of small intestinal crypts in Dom and Sub mice. (**K**,**L**) The mRNA expression levels of *ZO−1* and *TFF−3* in small intestinal tissues of mice were examined by qRT−PCR. Statistically significant differences are indicated: Student’s *t* test, n = 4 in Dom group, n = 12 in Sub group, * *p* < 0.05.

Next, Dom and Sub mice were orally gavaged with antibiotic cocktail (ABX) for 14 continuous days to eliminate enteric microorganisms before TAI (Figure 3A). Tube testing showed that ABX treatment induced a temporary disturbance in social hierarchy which was rehabilitated progressively after ABX removal (Figure 3B, Figure S3A). Intriguingly, ABX intervention erased the differences in intestinal radiation toxicity between Dom and Sub mice, as judged by similar colon length, inflammatory status, small intestine structure, and intestinal integrity (Figure 3C–L, Figure S3B). These findings hint that the gut microbiota of Dom mice might be the key factor eliciting intolerance of hosts to radiation stimuli.

**Figure 4:**
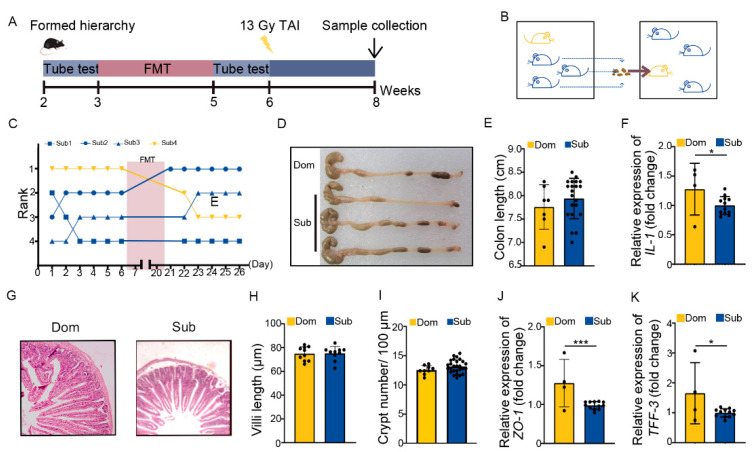
Transplanting gut microbiota from Sub mice to their Dom mouse alleviated intestinal radiation injuries. (**A**) Experimental protocol. (**B**) Schematic of FMT. (**C**) The rank positions of one cage of mice tested daily before and after FMT treatment. (**D**) Photographs of dissected colons from mice in the two groups. (**E**) Statistical comparison of colon length between the two groups. (**F**) The mRNA levels of IL−1 in small intestinal tissues were examined by qRT−PCR. (**G**) The morphology of small intestine was revealed by H&E staining. (**H**) Length of small intestinal villi in Dom and Sub mice. (**I**) Numbers of small intestinal crypts in Dom and Sub mice. (**J**,**K**) The mRNA expression levels of *ZO−1* and *TFF−3* in small intestinal tissues were examined by qRT−PCR. Statistically significant differences are indicated: Student’s *t* test, n = 4 in Dom group, n = 12 in Sub group, * *p* < 0.05 and *** *p* < 0.001.

The ABX administration mainly eliminated the gut microbes of hosts. We transplanted fecal microbiota from Sub mice to their respective Dom counterparts for 14 days before TAI to modify the gut microbiota pattern of high-ranking mice (Figure 4A,B). Fecal microbiota transplantation (FMT) degraded and maintained the social status of Dom mice more stably than ABX intervention (Figure 4C). In line with the ABX experiments, FMT not only lessened intestinal inflammatory status in Dom mice (Figure 4D–F, Figure S4A), but also improved their intestine structure and integrity (Figure 4G–K, Figure S4B). Together, ABX and FMT experiments suggest that differences in intestinal radiation toxicity induced by social hierarchy might depend at least partly on gut microbiota.

### 2.4. Lactobacillus Murinus Ameliorates Intestinal Radiation Injuries in Non-Social Hierarchy Mice

**Figure 5:**
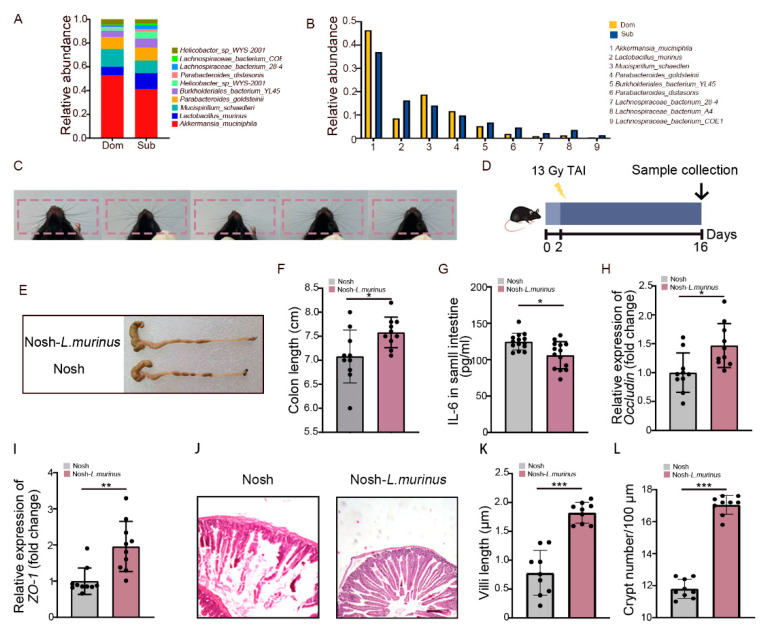
*L. murinus* alleviated intestinal radiation toxicity in mice without social hierarchy. (**A**,**B**) The relative abundances of the top 10 different strains of bacteria at the genus level in Dom and Sub mice after exposure to 13 Gy TAI. (**C**) Whiskers of five random mice in a group. (**D**) Experimental protocol. (**E**) Photographs of dissected colons. (**F**) Statistical comparison of colon length between the two groups. (**G**) Protein levels of IL-6 in the small intestine were measured by ELISA. (**H**,**I**) The mRNA expression levels of *occludin* and *ZO-1* in small intestinal tissues from the two groups were examined by qRT-PCR. (**J**) The morphology of the small intestine was revealed by H&E staining. (**K**) Small intestinal villi length. (**L**) Numbers of small intestinal crypts. Statistically significant differences are indicated: Student’s *t* test, n = 10 per group, * *p* < 0.05, ** *p* < 0.01 and *** *p* < 0.001.

Next, we aimed to screen for the gut microbe which was able to mitigate intestinal radiation injury in high-ranking mice. For the monoassociation study, *Lactobacillus murinus* (*L. murinus*) was selected due to (i) its recognition as a classic probiotic [35,36], (ii) its presence as the dominant gut strain in both Dom and Sub mice (Figure 1F and Figure 5A,B), (iii) its significant reduction in frequency in Dom mice after TAI (Figure S5A). We clarified the radioprotective function of *L. murinus* in mice without social hierarchy, which have been most frequently used in traditional research (Figure 5C). We treated the experimental mice with *L. murinus* orally once a day for 2 weeks before TAI (Figure 5D). As expected, mice receiving total abdominal irradiation that were gavaged with *L. murinus* had longer colons than the controls (Figure 5E,F). In addition, treatment with *L. murinus* alleviated inflammatory status (Figure 5G, Figure S5B–E) and up-regulated integrity-related genes (Figure 5H,I, Figure S5F) in small intestinal tissues. H&E staining further showed that *L. murinus* repaired the structure of the small intestine, as judged by taller and denser villi as well as more crypts retained (Figure 5J–L). Together, our observations indicate that *L. murinus* was able to alleviate intestinal radiation toxicity in mice without social hierarchy.

### 2.5. The Radioprotection of L. murinus Is Not Obvious for Dom Mice

**Figure 6:**
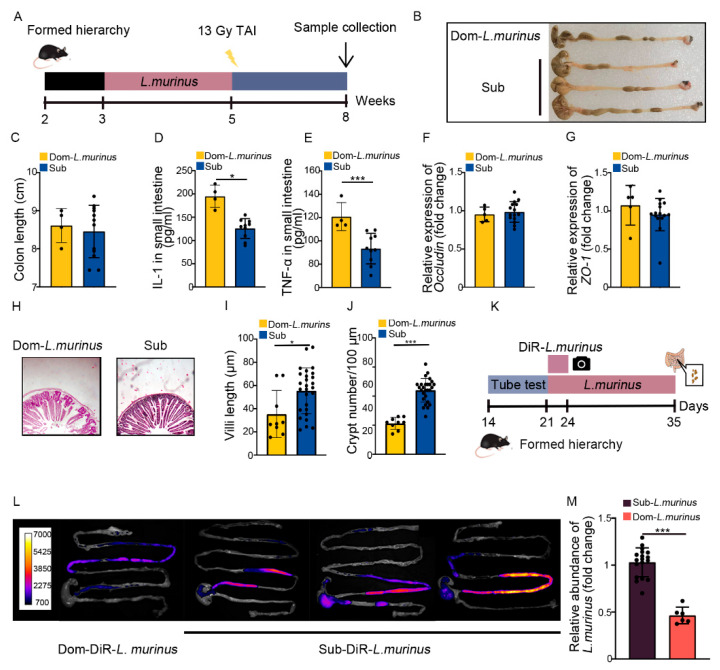
Radioprotection from *L. murinus* is not obvious for Dom mice. (**A**) Experimental protocol. (**B**) Photographs of dissected colons. (**C**) Statistical comparison of colon lengths between the two groups. (**D**,**E**) The protein levels of IL−1 and TNF−α in the small intestine were measured by ELISA. (**F**,**G**) The mRNA expression levels of *occludin* and *ZO−1* in small intestinal tissues from Dom mice treated with *L. murinus* and Sub mice were examined by qRT−PCR. (**H**) The morphology of the small intestine was revealed by H&E staining. (**I**) Length of villi in small intestinal tissues. (**J**) Numbers of crypts in small intestinal tissues. (**K**) Experimental protocol. (**L**) In vivo fluorescence imaging performed on Dom and Sub mice after DiR−labeled *L. murinus* administration. (**M**) Relative abundance in small intestines of Dom and Sub mice after *L. murinus* administration, according to q−PCR. Statistically significant differences are indicated: Student’s *t* test, n = 4 in Dom group, n = 12 in Sub group, * *p* < 0.05 and *** *p* < 0.001.

Next, we treated Dom mice with *L. murinus* using the aforementioned strategy (Figure 6A). Although Dom mice with *L. murinus* treatment had similar colon lengths (Figure 6B,C), the inflammatory status in the small intestine was higher than irradiated Sub mice, as judged by high levels of multiple inflammatory factors (Figure 6D,E, Figure S6A–C). *L. murinus* replenishment up-regulated the expression of integrity-related genes in Dom mice (Figure 6F,G, Figure S6D), but the villi remained scarce and short, accompanied by fewer crypts in the small intestine (Figure 6H–J). These results suggest that *L. murinus* cannot offer obvious therapeutic efficacy against intestinal radiation toxicity in Dom mice. To explore the underlying mechanism, Dom and Sub mice were orally gavaged with fluorescence-labeled *L. murinus* for 3 days. Then, the small and large intestines were collected and imaged by in vivo imaging (Figure 6K), and the fluorescence signals in intestines from Dom mice were faint compared with those from Sub mice (Figure 6L). We also collected the small intestinal contents from Dom and Sub mice after 14 days of *L. murinus* addition. q-PCR assay showed that the relative abundance of *L. murinus* was reduced in Dom mice compared with Sub mice (Figure 6M). The evidence indicates that *L. murinus* might not colonize stably in the GI tracts of higher status mice.

### 2.6. A. Muciniphila Colonizes in GI Tract Stably to Mitigate Intestinal Radiation Injury in Dom Mice

**Figure 7:**
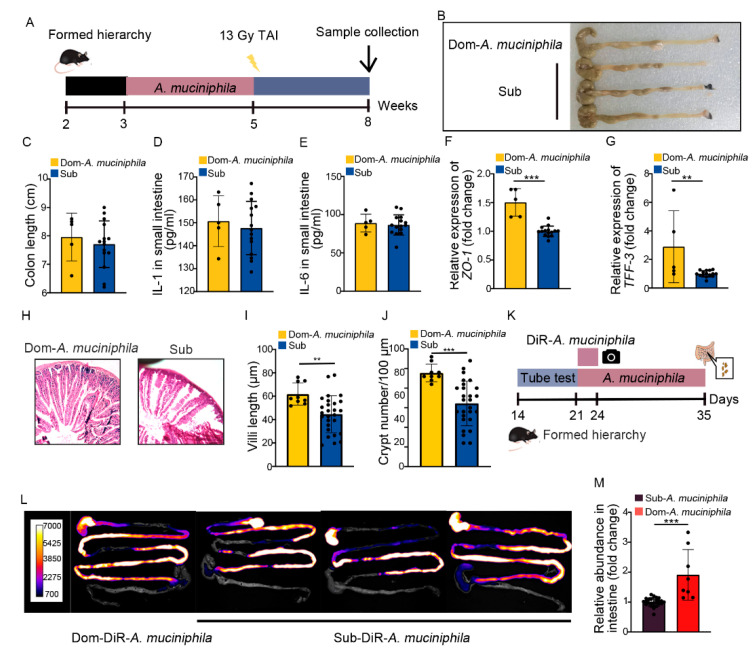
*A. muciniphila* stably colonizes in the GI tracts of Dom mice to mitigate intestinal radiation injury. (**A**) Experimental protocol. (**B**) Photographs of dissected colons. (**C**) Statistical comparison of colon lengths between the two groups. (**D**,**E**) The protein levels of IL−1 and IL−6 in the small intestine were measured by ELISA. (**F**,**G**) The mRNA expression levels of *ZO−1* and *TFF−3* in small intestinal tissues from Dom mice treated with *A. muciniphila* and Sub mice were examined by qRT−PCR. (**H**) The morphology of the small intestine was revealed by H&E staining. (**I**) Length of villi in small intestinal tissues. (**J**) Numbers of crypts in small intestinal tissues. (**K**) Experimental protocol. (**L**) In vivo fluorescence imaging performed on Dom and Sub mice given DIR−labeled *A. muciniphila*. (**M**) The relative abundance of *A. muciniphila* in small intestines of Dom and Sub mice was examined by q−PCR. Statistically significant differences are indicated: Student’s *t* test, n = 4 in Dom group, n = 12 in Sub group, ** *p* < 0.01 and *** *p* < 0.001.

Our previous study identified that gut *Akkermansia muciniphila* (*A. muciniphila*) was able to combat intestinal radiation injury in mice without social hierarchy [37]. In this study, *A. muciniphila* showed higher abundance and its frequency was less reduced than *L. murinus* in Dom mice (Figure S7A), implying that *A. muciniphila* might be the most prominent of gut microbiota in high-ranking mice. Thus, we treated Dom mice with *A. muciniphila* once a day for 2 weeks, by the same method as *L. murinus* replenishment (Figure 7A). Compared to the irradiated Sub mice, *A. muciniphila* lengthened the colon (Figure 7B,C) and decreased the inflammatory status of small intestinal tissues in Dom mice (Figure 7D,E, Figure S7B–E). *A. muciniphila* also up-regulated the expression of integrity-related genes and improved the structure of the small intestine (Figure 7F–J, Figure S7F). These results indicate that *A. muciniphila* bestowed better therapeutic efficacy than *L. murinus* in Dom mice. We also assessed the retention of *A. muciniphila* in Dom mice. Compared to *L. murinus*, *A. muciniphila* showed stabler colonization in the GI tracts of Dom mice (Figure 7L,M). Together, our observations indicate that *A. muciniphila* might be a more suitable probiotic for high-ranking mice to mitigate intestinal radiation toxicity.

## 3. Discussion

Owing to the rapid self-renewal of the epithelium, the small intestine is particularly sensitive and vulnerable to irradiation. Radiation exposure introduces massive cell death, represented by villi loss and epithelial barrier dysfunction [37]. Thus, pelvic and abdominal cancer patients present intestinal complications, such as enteritis, colitis, intestinal obstruction, anorexia, vomiting, etc., during and after radiotherapy. The development and treatment of intestinal radiation injuries have been widely investigated in clinical trials and pre-clinical animal models. In traditional animal research, animals have always been used without consideration being given to the establishment of social dominance hierarchy. However, establishing social hierarchies through competition and rivalry is a common behavior in animal and human societies [38]. Social hierarchy formation involves the activation of neural and endocrine systems [2], associated with the physiology and health of hosts [39] as well as the occurrence of multiple diseases including adiposity [40]. As a common refractory complication connected with radiation therapy in clinical scenarios, it has not been established whether and how intestinal radiation toxicity is influenced by social dominance hierarchy. In the present study, we housed four male mice in one cage for sufficient time to establish a natural social hierarchy and identified the high- and low-ranking mice. Although the body weight in the cohort was similar, the dominant mouse experienced lower survival rate and more serious intestinal injuries than the three submissive mice after the same doses of TAI. How did this interesting event occur?

Our previous studies identified that the gut microbiome is involved in radiation toxicity in hosts. Radiation-induced dysbiosis of the intestinal microecology can further promote radiogenic tissue damage [41]. Recent research has reported that high- or low-ranking mice harbor characteristic gut microbiota signatures. This suggests that the specific variations of gut microbiota between high- and low-ranking mice might be the key reason for the difference in the degree of intestinal radiation injuries. To verify our hypothesis, we analyzed the shifts of gut microbiota in Dom and Sub mice following TAI. Based on the caecotrophy of rodents, it has been observed that mice housed for a long time in the same cage share uniform intestinal microbiota [42]. The absence of significant differences between Dom and Sub mice before radiation exposure in terms of α- and β-diversity of gut microbiota supports this observation. However, the gut microbiota composition, including the dominant strains and their relative abundance, was clearly different between Dom and Sub mice. That might be the reason dominant mice showed differences in radiosensitivity compared with subordinates. We used deletion and restructure strategies to shape the gut microbiota in experimental mice. Firstly, ABX treatment not only deleted gut microbes but also erased the differences in radioactive intestinal injury between high- and low-ranking mice. Given that ABX might be a potential risk for the occurrence of inflammatory bowel disease [43], we further performed fecal microbiota transplantation. As expected, Dom mice carrying Sub mice’s fecal microbiota showed similar levels of intestinal radiation toxicity compared with their Sub companions. Together, the findings strengthen our hypothesis that gut microbiota play an important role in differences in intestinal radiation injury, and their mediation by social hierarchy. Considering the causes of radiosensitivity in Dom mice, the gut microbiota pattern differs between dominant and subordinate mice. According to our observations, the gut microbiota in Dom mice are more susceptible to irradiation, and the local abdominal irradiation-derived changes in dominant and subordinate mice show differences. However, the question requires further study. Notably, ABX treatment and FMT both affected the social hierarchy of the mouse colony, although FMT caused stabler changes than ABX. According to our findings, ABX gavage eliminated the majority of bacterial species in a short time, and the community was restored after ABX removal. However, FMT supplied a novel fecal microbiome which colonized in the GI tracts of receipts and reshaped their gut microbiota configuration, with the migratory organisms settling in the guts of recipients and having a longer lasting effect.

Considering the use of microbial preparations to mitigate intestinal radiation injury in Dom mice, it is relevant that 16S rRNA sequencing showed a significant reduction in the abundance of *L. murinus* among their prominent gut bacteria (Figure S4A), while levels remained basically unchanged in the case of *A. muciniphila* (Figure S5A). Both of these bacteria are regarded as potential probiotics in multiple scenarios, and our present and previous studies have identified that *L. murinus* and *A. muciniphila* were able to mitigate intestinal radiation injury in mouse models without hierarchical ranking. Thus, we focused on *L. murinus* and *A. muciniphila* as research objects to estimate their radioprotective effects in Dom mice. Intriguingly, only *A. muciniphila* demonstrated visible radioprotection in Dom mice, while that bestowed by *L. murinus* was negligible. These results indicate that not all microbial preparations applied to non-hierarchical mouse models are also suitable for the hierarchical colony. Foreign probiotics can interact with locally entrenched microbiome niches, which vary in their physiological properties along the GI tract, thus differently influencing host physiology [40]. *A. muciniphila* demonstrated stronger residual persistence than *L. murinus* in the GI tracts of Dom mice, hinting that the indigenous gut microecology profoundly impacted the foreign microbes. Returning to our 16S rRNA sequencing, two phenomena attracted our attention: (i) The relative abundance of *A. muciniphila* was much higher than that of *L. murinus* in the intestines of Dom mice without any stress. (ii) The frequency of *L. murinus* decreased significantly in Dom mice following radiation exposure, but that of *A. muciniphila* reduced only slightly. This suggests that the gut microhabitats of Dom mice might be more appropriate for *A. muciniphila*, which might be why *A. muciniphila* demonstrated stable colonization and overt radioprotective function in Dom mice.

The present study also provides some indications for the clinical translation of this basic research. For pre-clinical investigations, most medical research has traditionally used non-hierarchical animal models and have overlooked the effects of social hierarchy. As a result, their potential for clinical translation has been limited and our understanding of the actual molecular mechanisms involved has been impacted. Our results imply that animal models closer to the natural state should be considered in pre-clinical investigations, in order to generate data relevant to patients’ living conditions. For clinical applications, our and studies and others have identified that *A. muciniphila* and *L. murinus* are promising probiotics with medicinal value for multiple diseases [44,45,46]. However, the two probiotics do not perform equivalently in terms of therapeutic efficacy for intestinal radiation toxicity in all experimental mice, especially within a hierarchical colony. In clinical scenarios, microbial preparations such as probiotics have been widely used but with mixed success. Our findings show that a probiotic might be not suitable for all recipients and that the gut microecology of the host is a key factor for the efficacy of oral microbial preparations. Thus, it is necessary to screen and select optimal microbial preparations for different patients based on their gut microhabitats, to achieve effective and precise treatment. The pertinent question is how to choose the suitable microbial preparation for each individual. Our study hints that dominant strains in the GI tract which are resistant to external stimulation are more adaptive to hosts’ intestinal microhabitats and exhibit stable colonization. In this regard, the dominant intestinal species of patients with resistance to stimuli might be a valid reference for selection of microbial preparations to improve the therapeutic efficacy of probiotics. 

## 4. Materials and Methods

### 4.1. Mice

Eight- to 10-week-old (around 22 g) male C57BL/6J mice were purchased from the Beijing Huafukang Bioscience Co., Inc. (Beijing, China), and housed in a specific-pathogen-free (SPF) animal facility at the Institute of Radiation Medicine (IRM), Chinese Academy of Medical Sciences (CAMS). Mice were kept under standard conditions (ambient temperature 22 ± 2 °C, air humidity 40–70%, and a 12/12-h light/dark cycle) with continuous access to a standard diet and water.

To allow the mice to form a social hierarchy, four mice were housed in a cage for 2 weeks. To obtain mice with no social hierarchy, five mice were housed in a cage and the mice in the same cohort were blended and re-separated every 2 days throughout the whole experiment, thereby avoiding the formation of social hierarchy. Animal experiments were performed according to the institutional guidelines of the Animal Care and Ethics Committee of IRM-PUMC, compliant with the National Institutes of Health Guide for the Care and Use of Laboratory Animals (the Ethical Approval number is IRM-DWLL-2021-279 and 6 April 2021).

### 4.2. Tube Test

The standard tube test for the adult mice was modified from the published literature [47]. It employed a transparent Plexiglas tube of 30 cm length and 3 cm inside diameter, a size just sufficient to permit one adult mouse to pass through without reversing its direction. For training, each mouse was released at alternating ends of the tube and ran through the tube, sometimes with the help of a plastic stick pushing at its back. Each mouse was given eight training trials on each of two successive days. Following this, animals were tested daily with three further training trials before the test trials. The mouse that was first pushed out of the tube and set all four paws outside the tube within 2 min was designated the “loser” of that trial. In rare cases when no mice were pushed out within 2 min, the tests were repeated.

Between each trial, the tube was cleaned with 75% ethanol. A round robin design was applied to the four mice housed in each cage to allow the six possible pairs to compete. Mice were tested against one another in this manner in consecutive trials, with each mouse starting at an alternate end of the tube for each trial.

Tube tests were performed continuously, until the ranks were stable for at least 4 continuous daily trials. Based on six days of tube-test rankings, mice were distinguished as dominant (Dom) or submissive (Sub).

### 4.3. Irradiation Study

A Gammacell-40 137 Cs irradiator (Atomic Energy of Canada Limited, Chalk River, ON, Canada) at a dose rate of around 0.9 Gy per minute was used for all experiments. Mice were anesthetized with 3.8% chloral hydrate injection intraperitoneally (around 240 μL per mouse) and treated with total abdominal irradiation (TAI). For gastrointestinal (GI) tract experiments, the anesthetized mice were exposed to 13 Gy γ-ray using a lead shielding so that the whole abdomen was irradiated and the other parts of the mouse were shielded. Control mice were sham-irradiated. For the survival study, the mice were exposed to 18 Gy γ-ray local abdominal irradiation. Control mice were sham-irradiated.

### 4.4. Food Intake and Fecal Collection

Before measurement of food intake and fecal collection, Dom and Sub mice were housed singly for 12 h. Then, the food intake was measured and formed feces were collected from cage bedding before irradiation and every 2 days after irradiation.

### 4.5. Antibiotic Cocktail (ABX) Experiment

For oral antibiotic treatment, Dom and Sub mice were gavaged with an antibiotic cocktail at 200 µL/day for 2 weeks. The antibiotic cocktail was a proportional mix of ciprofloxacin (125 mg/L, Sigma-Aldrich, Madrid, Spain), metronidazole (100 mg/L, Sigma-Aldrich, Madrid, Spain), vancomycin (50 mg/L, Sigma-Aldrich, Madrid, Spain), streptomycin (100 U/L, Solarbio, Beijing, China), and penicillin (100 U/L, Solarbio, Beijing, China). Fresh antibiotic solution was prepared every day to ensure its activity.

### 4.6. Fecal Microbiota Transplantation Experiment

Fecal microbiota transplantation (FMT) was performed in each of the social hierarchy mice. According to convention, the Sub mice were used as donors to collect gut microbiota for the Dom mouse in the same cage. The donor’s fecal pellets were collected under SPF conditions. Donor stool was freshly prepared on the day of transplantation, and in all cases was prepared and transplanted within 4 h. Donor stool was weighed and diluted with 1 mL of saline per 0.1 g of stool. The stool was steeped in saline for about 15 min, shaken, and then centrifuged at 800 rpm for 3 min. The supernatant was obtained for treatment. Two hundred µL of the suspension was transferred by oral gavage.

### 4.7. Bacteria Strain Administration

*L. murinus* (CGMCC 1.2306) was cultured in MRS broth under strict anaerobic conditions. *A. muciniphila* MucT (ATCC BAA-835) was cultured in brain–heart infusion broth containing 10 mg/L resazurin (an oxidation–reduction indicator) under strict anaerobic conditions. A representative culture stock was used to determine the CFU/mL under anaerobic conditions, by plate counting using MRS agarose medium for *L. murinus*, and mucin media containing 1% agarose for *A. muciniphila*. This culture was diluted with anaerobic phosphate-buffered saline (PBS) to a final concentration of 1.5 × 10^8^ CFU/100 μL. Mice were gavaged with an oral administration of *L. murinus* or *A. muciniphila* (1.5 × 10^8^ CFU) suspended in sterile PBS for 14 days, while the contrast mice received sterile PBS with equivalent volume.

### 4.8. Ex Vivo Fluorescence Imaging

*L. murinus* (OD600 = 0.8) suspended in 1 mL anaerobic PBS was co-incubated with 20 μM DIR (dissolved in 5 μL DMSO) at 37 °C for 24 h, and *A. muciniphila* (OD600 = 0.8) suspended in 1 mL anaerobic PBS was co-incubated with 20 μM DIR (dissolved in 5 μL DMSO) at 37 °C for 1 h. The labeled bacteria were washed three times with anaerobic PBS to remove residual DIR, then resuspended with anaerobic PBS following centrifugation (3000 rpm, 5 min). DIR-labeled *L. murinus* or *A. muciniphila* was administrated orally to Dom and Sub mice at a concentration of 1 × 10^8^ CFU/200 μL per mouse. Then, the mice were sacrificed and the intestines were rapidly removed and stored at 4 °C in the dark. Ex vivo imaging was used for detecting *L. murinus* (in vivo master at 635–690 nm with exposure time 20 s) and *A. muciniphila* (in vivo master at 635–690 nm with exposure time 10 s) in dissected intestines. Data acquisition and analyses were performed with built-in software.

### 4.9. Quantitative Analysis of Bacteria

For the quantitative analysis of *L. murinus* and *A. muciniphila*, the small intestines of mice were cut lengthwise, and feces and mucus were scraped off with a sterile cotton brush. Then, the DNA was extracted using a TIANamp Stool DNA kit (TIANGEN, Beijing, China) and used for q-PCR. The q-PCR was performed according to instructions with Fast Start Universal SYBR Green Master (Rox) (Roche Diagnostics GmbH, Mannheim, Germany). Experiments were conducted in duplicate in three independent assays. Relative transcriptional folds were calculated as 2^−ΔΔCt^. Bacterial universal was used as a control for normalization. The primers are listed in Supplementary Table S1.

### 4.10. RNA Isolation and Quantitative Reverse Transcription Real-Time PCR

Total RNA was extracted from intestinal tissues, using TRIzol reagent (Invitrogen, Carlsbad, CA, USA) according to the manufacturer’s protocol. Complementary DNA was synthesized from total RNA using poly(A)-tailed total RNA and reverse transcription primer with ImPro-II Reverse Transcriptase (Promega, Madison, WI, USA), according to the manufacturer’s protocol. The qRT-PCR was performed according to instructions, using Fast Start Universal SYBR Green Master (Rox) (Roche Diagnostics GmbH, Mannheim, Germany). Experiments were conducted in duplicate in three independent assays. Relative transcriptional folds were calculated as 2^−ΔΔCt^. GAPDH was used as an internal control for normalization. All primers are listed in Supplementary Table S1.

### 4.11. Enzyme-Linked Immunosorbent Assay (ELISA)

Frozen intestinal samples were ground, and respectively reconstituted in PBS to a final concentration of 0.1 g/300 uL, followed by centrifuging for 10 min at 12,000× *g* and 4 °C. Protein levels were measured from the clear supernatant using an ELISA kit (Tongwei, Hefei, China) according to the manufacturer’s protocol. Optical density was read at 450 nm (Rayto, Shenzhen, China).

### 4.12. Histology

Following euthanasia, the small intestines of mice were fixed in 4% paraformaldehyde overnight at room temperature and then embedded in paraffin. Tissues were sectioned at 4 μm thickness and dipped in hematoxylin and eosin (H&E), using standard protocols. For the measurement of villus height, at least 15 well-oriented intact villus units were estimated for each mouse, using Image J 1.51 software. For the measurement of crypt numbers, 100 μm of small intestine tissue was used for counting crypt-villus units. No fewer than three crypt-villus units were counted for each Dom mouse, and at least nine for the contrasted Sub mice.

### 4.13. Bacterial Diversity Analysis

Stool samples were freshly collected from Dom and Sub mice in each cage and stored at −80 °C until use. Fresh feces were collected before radiation and at 14 d after radiation, respectively, for downstream analysis. DNA was extracted from the stool samples using the PowerFecal^®^ DNA Isolation Kit (MoBio Carlsbad, California, USA) before radiation or after 14 d of radiation. The 16S ribosomal RNA (rRNA) V4 gene amplification and sequencing were carried out using Illumina MiSeq technology. Sequence analyses were performed by Uparse software (Uparse v7.0.1001, http://drive5.com/uparse/, 7 June 2022). Sequences with ≥97% similarity were assigned to the same OTUs. The representative sequence for each OTU was screened for further annotation. For each representative sequence, the Silva123 Database was used based on the RDP classifier algorithm (Version 2.2, http://sourceforge.net/projects/rdp-classifier/, 7 June 2022) to annotate taxonomic information. The primers are listed in Supplementary Table S1.

### 4.14. Statistical Analysis

Each experiment was repeated at least three times. The data are presented as the means ± SD with respect to the number of samples (n) in each group. Significance was assessed by comparing the mean values using Student’s *t*-test for independent groups: * *p* < 0.05; ** *p* < 0.01; and *** *p* < 0.005. Results with *p* < 0.05 were considered statistically significant.

## Data Availability

Not applicable.

## Supplementary Materials

The following supporting information can be downloaded at: https://www.mdpi.com/article/10.3390/ijms232113189/s1.

## References

[1] Chou Y.J., Ma Y.K., Lu Y.H., King J.T., Tasi W.S., Yang S.B., Kuo T.H. (2022). Potential cross-species correlations in social hierarchy and memory between mice and young children. Commun. Biol..

[2] Wang Y., Dai G., Gu Z., Liu G., Tang K., Pan Y.H., Chen Y., Lin X., Wu N., Chen H. (2020). Accelerated evolution of an Lhx2 enhancer shapes mammalian social hierarchies. Cell Res..

[3] Wang F., Kessels H.W., Hu H. (2014). The mouse that roared: Neural mechanisms of social hierarchy. Trends Neurosci..

[4] Smith C.R., Toth A.L., Suarez A.V., Robinson G.E. (2008). Genetic and genomic analyses of the division of labour in insect societies. Nat. Rev. Genet..

[5] Evans O., Rodriguez-Borillo O., Font L., Currie P.J., Pastor R. (2020). Alcohol Binge Drinking and Anxiety-Like Behavior in Socialized Versus Isolated C57BL/6J Mice. Alcohol. Clin. Exp. Res..

[6] Fournier M.A. (2020). Dimensions of human hierarchy as determinants of health and happiness. Curr. Opin. Psychol..

[7] Gospocic J., Glastad K.M., Sheng L., Shields E.J., Berger S.L., Bonasio R. (2021). Kr-h1 maintains distinct caste-specific neurotranscriptomes in response to socially regulated hormones. Cell.

[8] Chagas L.A., Batista T.H., Ribeiro A., Ferrari M.S., Vieira J.S., Rojas V., Kalil-Cutti B., Elias L., Giusti-Paiva A., Vilela F.C. (2021). Anxiety-like behavior and neuroendocrine changes in offspring resulting from gestational post-traumatic stress disorder. Behav. Brain Res..

[9] Louch C.D., Higginbotham M. (1967). The relation between social rank and plasma corticosterone levels in mice. Gen. Comp. Endocrinol..

[10] Palle A., Zorzo C., Luskey V.E., McGreevy K.R., Fernandez S., Trejo J.L. (2019). Social dominance differentially alters gene expression in the medial prefrontal cortex without affecting adult hippocampal neurogenesis or stress and anxiety-like behavior. FASEB J..

[11] Stagkourakis S., Spigolon G., Williams P., Protzmann J., Fisone G., Broberger C. (2018). A neural network for intermale aggression to establish social hierarchy. Nat. Neurosci..

[12] Ma M., Xiong W., Hu F., Deng M.F., Huang X., Chen J.G., Man H.Y., Lu Y., Liu D., Zhu L.Q. (2020). A novel pathway regulates social hierarchy via lncRNA AtLAS and postsynaptic synapsin IIb. Cell Res..

[13] Huntingford F.A., Turner A.K. (1987). The consequences of animal conflict. Animal Conflict.

[14] LeClair K.B., Chan K.L., Kaster M.P., Parise L.F., Burnett C.J., Russo S.J. (2021). Individual history of winning and hierarchy landscape influence stress susceptibility in mice. eLife.

[15] WARREN S., BOWERS J.Z. (1950). The acute radiation syndrome in man. Ann. Intern. Med..

[16] Weisdorf D., Chao N., Waselenko J.K., Dainiak N., Armitage J.O., McNiece I., Confer D. (2006). Acute radiation injury: Contingency planning for triage, supportive care, and transplantation. Biol. Blood Marrow Transplant..

[17] Nag D., Bhanja P., Riha R., Sanchez-Guerrero G., Kimler B.F., Tsue T.T., Lominska C., Saha S. (2019). Auranofin Protects Intestine against Radiation Injury by Modulating p53/p21 Pathway and Radiosensitizes Human Colon Tumor. Clin. Cancer Res..

[18] Riehl T.E., Alvarado D., Ee X., Zuckerman A., Foster L., Kapoor V., Thotala D., Ciorba M.A., Stenson W.F. (2019). Lactobacillus rhamnosus GG protects the intestinal epithelium from radiation injury through release of lipoteichoic acid, macrophage activation and the migration of mesenchymal stem cells. Gut.

[19] Wang Z., Chen Z., Jiang Z., Luo P., Liu L., Huang Y., Wang H., Wang Y., Long L., Tan X. (2019). Cordycepin prevents radiation ulcer by inhibiting cell senescence via NRF2 and AMPK in rodents. Nat. Commun..

[20] Carmody R.N., Sarkar A., Reese A.T. (2021). Gut microbiota through an evolutionary lens. Science.

[21] Weersma R.K., Zhernakova A., Fu J. (2020). Interaction between drugs and the gut microbiome. Gut.

[22] Matenchuk B.A., Mandhane P.J., Kozyrskyj A.L. (2020). Sleep, circadian rhythm, and gut microbiota. Sleep Med. Rev..

[23] Lee M., Chang E.B. (2021). Inflammatory Bowel Diseases (IBD) and the Microbiome-Searching the Crime Scene for Clues. Gastroenterology.

[24] Tanes C., Bittinger K., Gao Y., Friedman E.S., Nessel L., Paladhi U.R., Chau L., Panfen E., Fischbach M.A., Braun J. (2021). Role of dietary fiber in the recovery of the human gut microbiome and its metabolome. Cell Host Microbe.

[25] Wang T., Xu J., Xu Y., Xiao J., Bi N., Gu X., Wang H.L. (2022). Gut microbiota shapes social dominance through modulating HDAC2 in the medial prefrontal cortex. Cell Rep..

[26] Chen S., Yan C., Liu W., Chen K., Xing L., Li H., Zhao X. (2022). Research Note: Integrated gut microbiome and short-chain fatty acids responds to dominance hierarchy in roosters. Poult. Sci..

[27] Luo Y., Zhao P., Dou M., Mao J., Zhang G., Su Y., Wang Q., Wang Q., Wang Y., Sun R. (2021). Exogenous microbiota-derived metabolite trimethylamine N-oxide treatment alters social behaviors: Involvement of hippocampal metabolic adaptation. Neuropharmacology.

[28] Xiao H.W., Cui M., Li Y., Dong J.L., Zhang S.Q., Zhu C.C., Jiang M., Zhu T., Wang B., Wang H.C. (2020). Gut microbiota-derived indole 3-propionic acid protects against radiation toxicity via retaining acyl-CoA-binding protein. Microbiome.

[29] Li Y., Dong J., Xiao H., Zhang S., Wang B., Cui M., Fan S. (2020). Gut commensal derived-valeric acid protects against radiation injuries. Gut Microbes.

[30] Xiao H.W., Li Y., Luo D., Dong J.L., Zhou L.X., Zhao S.Y., Zheng Q.S., Wang H.C., Cui M., Fan S.J. (2018). Hydrogen-water ameliorates radiation-induced gastrointestinal toxicity via MyD88’s effects on the gut microbiota. Exp. Mol. Med..

[31] Cui M., Xiao H., Li Y., Zhang S., Dong J., Wang B., Zhu C., Jiang M., Zhu T., He J. (2019). Sexual Dimorphism of Gut Microbiota Dictates Therapeutics Efficacy of Radiation Injuries. Adv. Sci..

[32] Wang B., Zhang S.Q., Dong J.L., Li Y., Jin Y.X., Xiao H.W., Wang H.C., Fan S.J., Cui M. (2022). Ambient temperature structures the gut microbiota of zebrafish to impact the response to radioactive pollution. Environ. Pollut..

[33] Fan Z., Zhu H., Zhou T., Wang S., Wu Y., Hu H. (2019). Using the tube test to measure social hierarchy in mice. Nat. Protoc..

[34] Hu J., Deng F., Zhao B., Lin Z., Sun Q., Yang X., Wu M., Qiu S., Chen Y., Yan Z. (2022). *Lactobacillus murinus* alleviate intestinal ischemia/reperfusion injury through promoting the release of interleukin-10 from M2 macrophages via Toll-like receptor 2 signaling. Microbiome.

[35] Wilck N., Matus M.G., Kearney S.M., Olesen S.W., Forslund K., Bartolomaeus H., Haase S., Mahler A., Balogh A., Marko L. (2017). Salt-responsive gut commensal modulates TH17 axis and disease. Nature.

[36] Wang B., Jin Y.X., Dong J.L., Xiao H.W., Zhang S.Q., Li Y., Chen Z.Y., Yang X.D., Fan S.J., Cui M. (2021). Low-Intensity Exercise Modulates Gut Microbiota to Fight Against Radiation-Induced Gut Toxicity in Mouse Models. Front. Cell Dev. Biol..

[37] Duhachek-Muggy S., Bhat K., Medina P., Cheng F., He L., Alli C., Saki M., Muthukrishnan S.D., Ruffenach G., Eghbali M. (2020). Radiation mitigation of the intestinal acute radiation injury in mice by 1-[(4-nitrophenyl)sulfonyl]-4-phenylpiperazine. Stem Cells Transl. Med..

[38] Hobson E.A., Monster D., DeDeo S. (2021). Aggression heuristics underlie animal dominance hierarchies and provide evidence of group-level social information. Proc. Natl. Acad. Sci. USA.

[39] van den Berg W.E., Lamballais S., Kushner S.A. (2015). Sex-specific mechanism of social hierarchy in mice. Neuropsychopharmacology.

[40] Russell G., Lightman S. (2019). The human stress response. Nat. Rev. Endocrinol..

[41] Gerassy-Vainberg S., Blatt A., Danin-Poleg Y., Gershovich K., Sabo E., Nevelsky A., Daniel S., Dahan A., Ziv O., Dheer R. (2018). Radiation induces proinflammatory dysbiosis: Transmission of inflammatory susceptibility by host cytokine induction. Gut.

[42] Caruso R., Ono M., Bunker M.E., Nunez G., Inohara N. (2019). Dynamic and Asymmetric Changes of the Microbial Communities after Cohousing in Laboratory Mice. Cell Rep..

[43] Virta L., Auvinen A., Helenius H., Huovinen P., Kolho K.L. (2012). Association of repeated exposure to antibiotics with the development of pediatric Crohn’s disease--a nationwide, register-based finnish case-control study. Am. J. Epidemiol..

[44] Yuan T., Wang J., Chen L., Shan J., Di L. (2020). *Lactobacillus murinus* Improved the Bioavailability of Orally Administered Glycyrrhizic Acid in Rats. Front. Microbiol..

[45] Qu S., Fan L., Qi Y., Xu C., Hu Y., Chen S., Liu W., Liu W., Si J. (2021). *Akkermansia muciniphila* Alleviates Dextran Sulfate Sodium (DSS)-Induced Acute Colitis by NLRP3 Activation. Microbiol Spectr.

[46] Wang L., Tang L., Feng Y., Zhao S., Han M., Zhang C., Yuan G., Zhu J., Cao S., Wu Q. (2020). A purified membrane protein from *Akkermansia muciniphila* or the pasteurised bacterium blunts colitis associated tumourigenesis by modulation of CD8(+) T cells in mice. Gut.

[47] Chou Y.J., Lu Y.H., Ma Y.K., Su Y.S., Kuo T.H. (2021). The decisive role of subordination in social hierarchy in weanling mice and young children. iScience.

