# Supplementary material for: Social Hierarchy Dictates Intestinal Radiation Injury in a Gut Microbiota-Dependent Manner
Source: Int J Mol Sci. 2022 Oct 29;23(21):13189. doi: 10.3390/ijms232113189 (PMC9659279; doi:10.3390/ijms232113189)
Supplement: Supplementary file 1 [file ijms-23-13189-s001.zip › ijms-1827915-supplementary.pdf]

Supplementary materials

1 Supplementary Tables

Table S1. All primers used in this experiment

Gene	Primer	Sequence (5′-3′)
**Primers for PCR**		
GAPDH	forward	TGTTTCCTCGTCCCGTAGA
	reverse	CAATCTCCACTTTGCCACTG
IL-1	forward	TTGAAGAAGAGCCCATCCTC
	reverse	CAGCTCATATGGGTCCGAC
IL-6	forward	TGTGCAATGGCAATTCTGAT
	reverse	GGTACTCCAGAAGACCAGAGGA
TNF-a	forward	TTCTCATTCCTGCTTGTGGCA
	reverse	ACTTGGTGGTTTGCTACGACG
TFF-3	forward	CTGGCTAATGCTGTTGGT
	reverse	TTGTTACACTGCTCCGATG
ZO-1	forward	CCCTACCAACCTCGGCCTT
	reverse	AACGCTGGAAATAACCTCGTTC
Occludin	forward	ATGTCCGGCCGATGCTCTC
	reverse	TTTGGCTGCTCTTGGGTCTGTAT
**Primers for sequencing**		
515F		GTGCCAGCMGCCGCGGTAA
806R		GGACTACHVGGGTWTCTAAT
**16S rRNA Primers for PCR**		
Bacterial universal	forward	ACTCCTACGGGAGGCAGCAG
	reverse	ATTACCGCGGCTGCTGG
A. muciniphila	forward	CAGCACGTGAAGGTGGGGAC
	reverse	CCTTGCGGTTGGCTTCAGAT
L. murinus	forward	AGAGTTTGATCCTGGCTCAG
	reverse	GGTTACCTTGTTACGACTT

Table S2. The name of gut bacteria at the genus level in Dom and Sub mice

1	GCA-900066575
2	Lachnospiraceae_NK4A136_group
3	Erysipelotrichaceae_UCG-003
4	Bifidobacterium
5	Escherichia-Shigella
6	Lachnoclostridium
7	Helicobacter
8	Dorea
9	Alloprevotella
10	Blautia
11	Mucispirillum

12	Lachnospiraceae_UCG-001
13	Monoglobus
14	Bacteroides
15	Oscillibacter
16	Colidextribacter
17	Lactobacillus
18	Alistipes
19	Ligilactobacillus
20	Prevotellaceae_NK3B31_group
21	Allobaculum
22	Limosilactobacillus
23	[Eubacterium]_xylanophilum_group
24	unidentified_Lachnospiraceae
25	[Eurbacterium]_fissicatena_group
26	Rikenella
27	Roseburia
28	Parasutterella
29	Dubosiella
30	Muribaculum
31	lleibacterium
32	Pseudomonas
33	Akkermansia
34	Prevotellaceae_UCG-001
35	Parabacteroides

Table S3. The name of gut bacteria at the genus level in Dom and Sub mice before and after radiation

1	Allobaculum
2	lleibacterium
3	Mucispirillum
4	Parabacteroides
5	Roseburia
6	A2
7	Ligilactobacillus
8	Lachnospiraceae_UCG-001
9	Candidatus_Saccharimonas
10	Limosilactobacillus
11	Unidentified_Lachnospiraceae
12	Rikenella
13	[Eubacterium]_xylanophilum_group
14	Lactobacillus
15	Muribaculum
16	Oscillibacter
17	Helicobacter

18	Alistipes
19	Blautia
20	Dubosiella
21	Akkermansia
22	ASF356
23	Prevotellaceae_NK4A136_group
24	Colidextribacter
25	Marinobacter
26	Leuconostoc
27	Bacterodies
28	Acetobacter
29	Parasutterella
30	Prevotellaceae_UCG-001
31	Prevotellaceae_NK3B31_group
32	Bifidobacterium
33	Alloprevotella
34	Pseudomonas
35	[Eubacterium]_fissicatena_group

2 Supplementary Figures

Figure S1

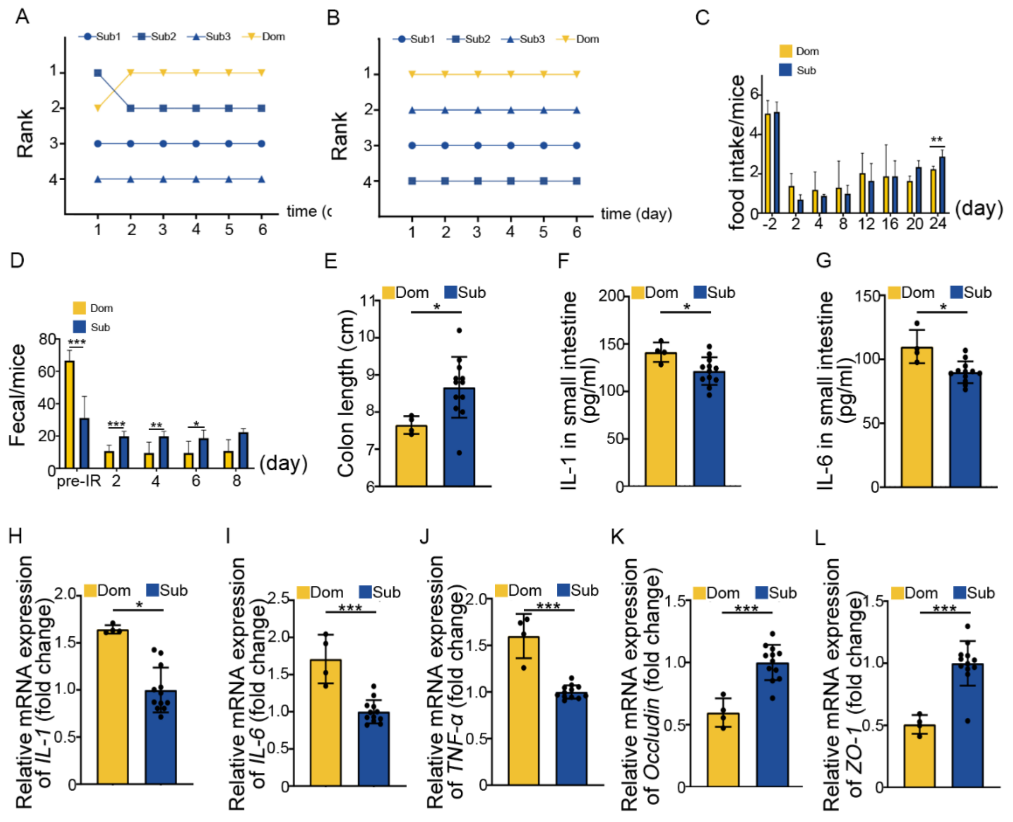

Figure S1. Social hierarchy determines the degree of radiation intestinal injury. (A and B) The rank

positions of one cage of mice tested daily over 6 days. (C) Food intake per mouse. (D) Number of formed fecal pelleted per mouse. (E) Colon length of Dom and Sub. (F and G) The protein levels of IL-1 and IL-6 in small intestine were measured by ELISA. (H-L) The mRNA expression levels of *IL-1*, *IL-6*, *TNF-α, Occludin* and *ZO-1* were examined in small intestine tissues from Dom and Sub mice by qRT-PCR. (Statistically significant differences are indicated: Student's *t* test, n=4 in Dom group, n=12 in Sub group, * $p < 0.05$, ** $p < 0.01$ and *** $p < 0.001$).

Figure S2

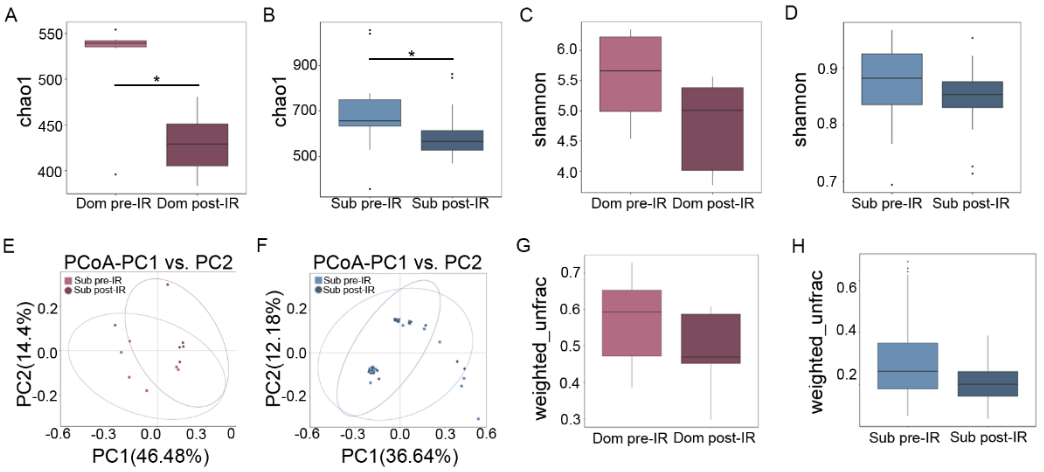

Figure S2. Gut bacteria community shifts significantly in Dom mice following irradiation. (A and B) The α-diversity of enteric bacteria of Dom mice and Sub mice before and after radiation was compared by chao1 index. (C and D) The α-diversity of enteric bacteria of Dom mice and Sub mice before and after radiation was compared by Shannon index. (Eand F) PCA were used to examine the alteration of intestinal bacteria taxonomic pattern. (G and H) The β-diversity of enteric bacteria of Dom mice (G) and Sub mice (H) was compared by weighted unifrac analysis. (Statistically significant differences are indicated: Student's *t* test, n=6 in Dom pre-IR and Dom post-IR group, n=18 in Sub pre-IR and Sub post-IR group, * $p < 0.05$, ** $p < 0.01$ and *** $p < 0.001$).

Figure S3

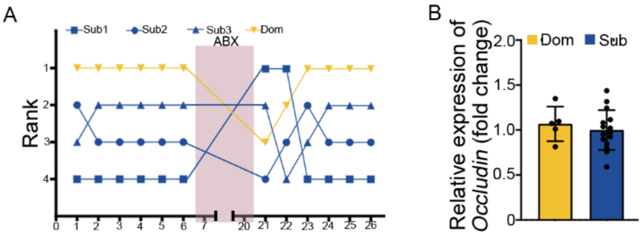

Figure S3. ABX treatment eliminated the difference in intestinal radiation injury between Dom and Sub mice. (A) The rank positions of one cage of mice tested daily before and after ABX treatment. (B) The mRNA expression levels of *Occludin* were examined in small intestine tissues from mice by qRT-PCR. (Statistically significant differences are indicated: Student's t test, n=10 per group, * $p < 0.05$, ** $p < 0.01$ and *** $p < 0.001$).

Figure S4

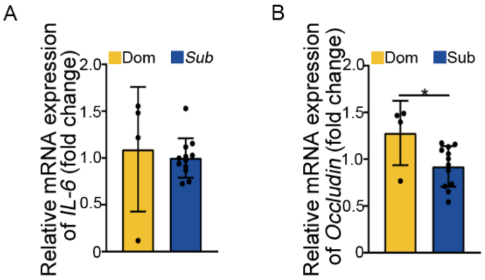

Figure S4. FMT treatment eliminated the difference in intestinal radiation injury between Dom and Sub mice. (A and B) The mRNA expression levels of *IL-6* and *Occludin* were examined in small intestine tissues from mice by qRT-PCR. (Statistically significant differences are indicated: Student's t test, n=10 per group, * p < 0.05, ** p < 0.01 and *** p < 0.001).

Figure S5

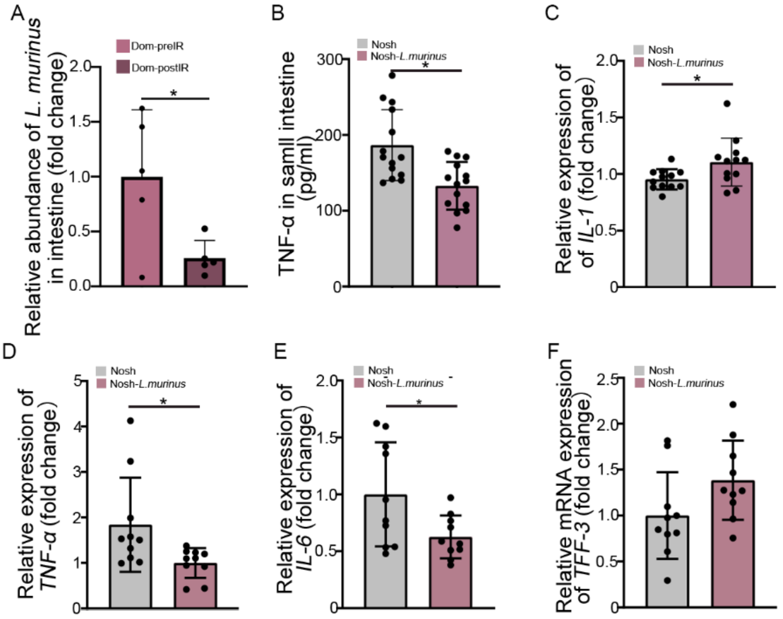

Figure S5 *L. murinus* is able to alleviate intestinal radiation toxicity in non-social hierarchy mice. (A) Relative abundance of *L. murinus* in small intestine of Dom mice before and after 13 Gy TAI. (B) The protein levels of TNF-α in small intestine were measured by ELISA. (C-F) The mRNA expression levels of *IL-1*, *TNF-α*, *IL-6* and *TFF-3* were examined in small intestine tissues by qRT-PCR. (Statistically significant differences are indicated: Student's *t* test, n=10 per group, * $p < 0.05$, ** $p < 0.01$ and *** $p < 0.001$).

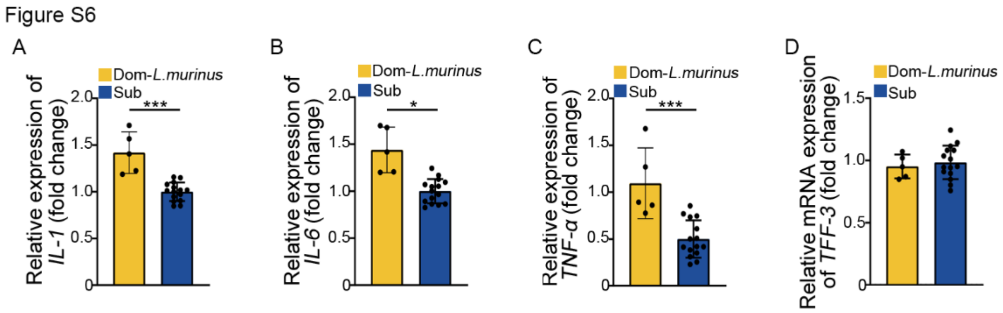

Figure S6. The radioprotection of *L. murinus* is not obvious for Dom mice. (A-D) The mRNA expression levels of *IL-1*, *IL-6*, *TNF-α* and *TFF-3* were examined in small intestine tissues by qRT-PCR. (Statistically significant differences are indicated: Student's *t* test, n=4 in Dom group, n=12 in Sub group, * $p < 0.05$, ** $p < 0.01$ and *** $p < 0.001$).

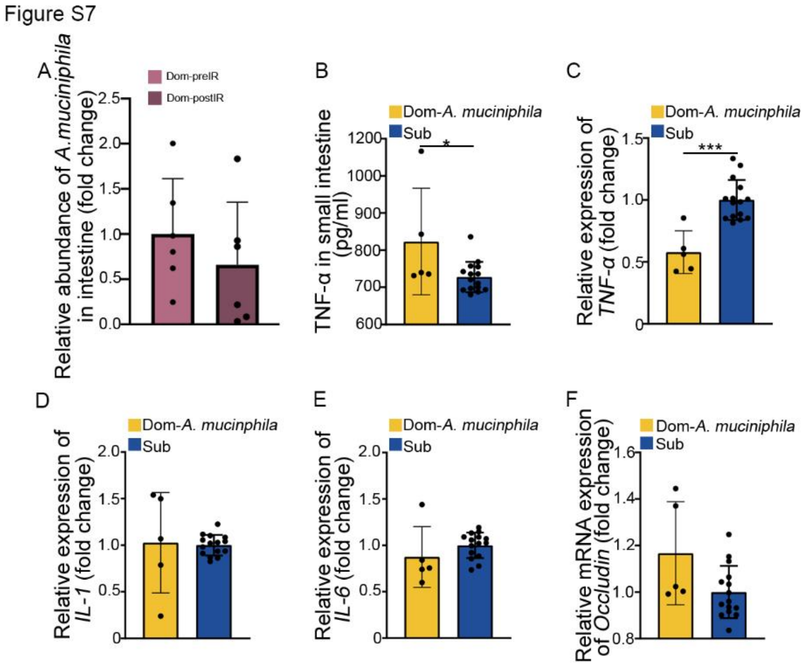

Figure S7. *A. muciniphila* colonizes in GI tract of Dom mice stably to mitigate intestinal radiation injury. (A) Relative abundance of *A. muciniphila* in small intestine of Dom mice before and after 13 Gy TAI. (B) The protein levels of TNF-α in small intestine were measured by ELISA. (C-F) The mRNA expression levels of *TNF-α*, *IL-1*, *IL-6* and *Occludin* were examined in small intestine tissues by qRT-PCR. (Statistically significant differences are indicated: Student's *t* test, n=4 in Dom group, n=12 in Sub group, * $p < 0.05$, ** $p < 0.01$ and *** $p < 0.001$).
